# Speciation within the *Anopheles gambiae* complex: high-throughput whole genome sequencing reveals evidence of a putative new cryptic taxon in ‘far-west’ Africa

**DOI:** 10.21203/rs.3.rs-3914444/v1

**Published:** 2024-03-18

**Authors:** B. Caputo, C.M. De Marco, V. Pichler, G. Bottà, K.L. Bennett, C.S. Clarkson, J.A. Tennessen, D. Weetman, A. Miles, A. della Torre

**Affiliations:** 1 Dipartimento di Sanità Pubblica e Malattie Infettive, Istituto Pasteur Italia-Fondazione Cenci-Bolognetti, Università di Roma “Sapienza”, Rome Italy; 2 Wellcome Sanger Genomic Surveillance Unit, Wellcome Sanger Institute, Hinxton, Cambridge, UK; 3 Harvard T.H. Chan School of Public Health, Boston, MA, USA; 4 Broad Institute, Cambridge, MA, USA; 5 Liverpool School of Tropical Medicine, Liverpool, UK

**Keywords:** Anopheles, malaria vectors, reproductive barriers, admixture, gene-flow

## Abstract

The two main Afrotropical malaria vectors - *Anopheles coluzzii* and *An. gambiae* – are genetically distinct and reproductively isolated across West Africa. However, populations at the western extreme of their range are assigned as “intermediate” between the two species by whole genome sequence (WGS) data, and as hybrid forms by conventional molecular diagnostics. By exploiting WGS data from 1,190 specimens collected across west Africa via the *Anopheles gambiae* 1000 Genomes network, we identify a novel putative taxon in the far-west (provisionally named Bissau molecular form), which did not arise by admixture but rather originated at the same time as the split between *An. coluzzii* and *An. gambiae*. Intriguingly, these populations lack insecticide resistance mechanisms commonly observed in the two main species. These findings lead to a change of perspective on malaria vector species in the far-west region with potential for epidemiological implications, and a new challenge for genetic-based mosquito control approaches.

## INTRODUCTION

Members of the *Anopheles gambiae* complex include the most important Afrotropical malaria vectors. Because of their huge public health relevance, they have been subject of cytogenetic, genetic, and genomic studies for more than 50 years. The large amount of knowledge and data available on these species puts them at the forefront of research on genomic patterns associated with species formation. *Anopheles gambiae* complex species have invaluable features for model organisms in the speciation genomics field: i) despite being morphologically indistinguishable, they are ecologically differentiated, particularly at the larval stage when they show adaptation to different breeding habitats^1–3^; ii) they are characterized by a short generation time (~1 generation/ month) enhancing opportunities to monitor genomic changes over time; iii) they show very large effective population sizes (Ne~5–6 million) and almost unparalleled nucleotide diversity^4,5^, enhancing capacity for adaptive evolution; iv) they occur over a broad distribution range, encompassing contrasting environments as well as potential geographical and anthropogenic barriers^6,7^ v) they have different degrees of reproductive isolation by post-mating isolation and/or pre-mating mechanisms^8^; vii) they are subject to strong and contemporary selection pressures due to extensive use of insecticides during the last 70 years, which has driven adaptive introgression^9,10^ and allows observation of genomic evolution in short ecological timescales.

Nine sibling species are presently known in the complex, three of which were only described in the last decade. *Anopheles coluzzii* and *An. gambiae* have been raised to the species status in 2013^11^ following evidence of genetic discontinuities within *An. gambiae* s.s. These were initially based on different inversion arrangements on the right arm of chromosome-2, which led to the description of five sympatric non-panmictic “chromosomal forms”, informally named MOPTI, SAVANNA, BAMAKO, FOREST and BISSAU^12,13^. Later, two assortative mating units (the M and S-molecular forms) partially overlapping with the SAVANNA and MOPTI chromosomal forms were described based on form-specific SNPs in the rDNA IGS region^14^. The availability of easy genotyping of these SNPs^15,16^ allowed evidence to accumulate on the genomic and ecological differentiation of the two forms eventually leading to the description of the two species^11^. The remaining chromosomal forms remain less clearly defined by contemporary population genomic approaches.

*Anopheles coluzzii* and *An. gambiae* are sympatric in sub-Saharan Africa west of the Rift Valley where they represent the major vectors of human malaria. The species’ ability to benefit from human-made habitats has linked their speciation process to human-made environmental changes. *Anopheles coluzzii* has broken free from the need to rely on rain-dependent breeding sites - typical of fresh-water species of the complex, including *An. gambiae* - by adapting to more permanent breeding sites produced by irrigation, deforestation and urbanization. While in the latter species intraspecific differentiation is extremely weak across the whole range, *An. coluzzii* shows a “bimodal” distribution pattern with populations occupying the xeric savanna belt in the northern hemisphere differentiated from populations occupying the ribbon of coastline along the Gulf of Guinea^17,18^.

*Anopheles coluzzii* and *An. gambiae* are known to mate freely and produce viable progeny under laboratory conditions. However, putative hybrids (i.e. individuals showing a heterozygous pattern of IGS diagnostic markers; hereafter putative IGS-hybrids) are rarely found at frequencies >0.2 in the field, due to – incompletely understood - pre-mating mechanisms and post-mating selection against hybrids^8^. However, in coastal areas at the western extreme of the two species range (hereafter, ‘far-west’) frequencies of putative IGS-hybrids >20% are stably observed^19,20^. Genetic studies have revealed that these ‘far-west’ populations are highly differentiated from neighbouring populations in inland areas^21–23^, leading to the hypothesis that they may have originated from extensive introgressive hybridization between the two species^23^. Based on Ag1000G Phase-1 and Phase-2 whole genome sequence (WGS) data, these coastal populations (which we refer to as *gcx1* and *gcx2*) were later defined as “intermediate” between *An. coluzzii* and *An. gambiae*^4,18^. Interestingly, seminal studies on paracentric inversion polymorphisms in the same geographical region led to the description of the BISSAU chromosomal form, characterised by high frequencies of 2Rd and low frequencies of 2La inversions^12,24^.

The aim of this work was to understand the actual nature of ‘far-west’ populations focusing on their relationships with west-central *An. coluzzii* and *An. gambiae* populations. Specifically, we examine the genetically distinct far west populations *gcx1* (comprising *gcx1-GM* populations from The Gambia, and *gcx1-GW* populations from Guinea Bissau) and *gcx2*. To this aim, we exploited Ag1000G Phase-3 data^25^, which include additional samples from The Gambia and Guinea Bissau compared to the ‘far-west’ Phase-1 and −2 populations. Results obtained by population genomic, divergence and demographic analyses illustrate the need for a change of perspective in the current view of ongoing evolutionary processes in major malaria vector species at the western extremes of Sub-Saharan Africa, suggesting the existence of a novel putative taxon diverged from *An. gambiae*. Available data on genetic traits associated with resistance to insecticides in this taxon suggest lack of current gene-flow with both *An. coluzzii* and *An. gambiae*.

## MATERIALS AND METHODS

### 1- West-African Ag1000G Phase-3 data

**Figure 1.**
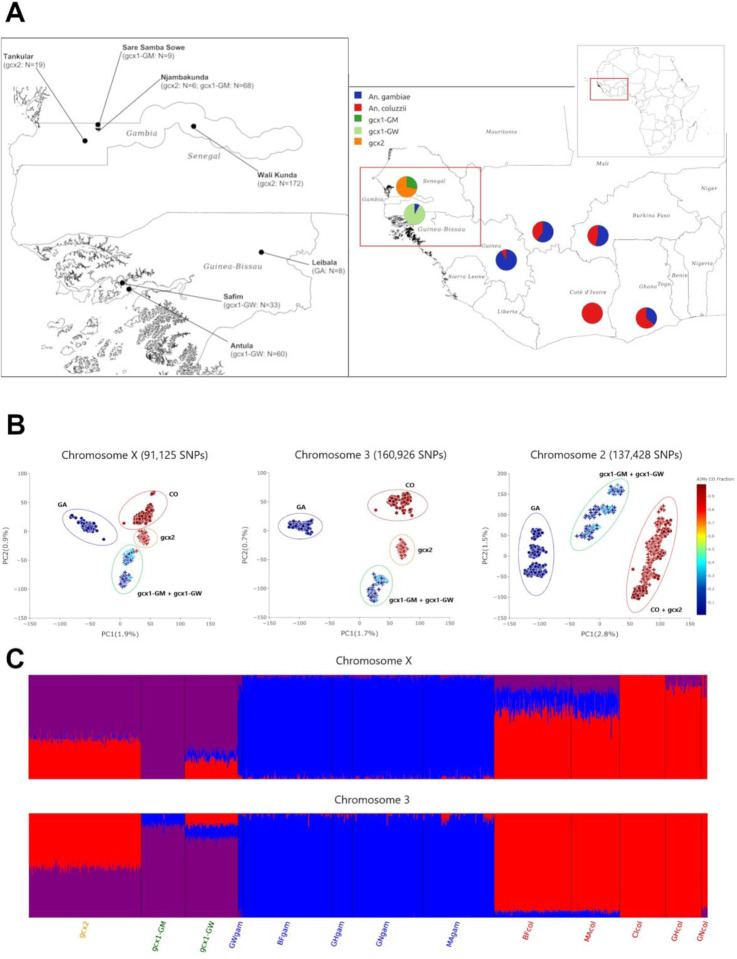
Genomic structure of Phase-3 Ag1000G *Anopheles gambiae* s.l. from West and Far-West Africa. **A -** Distribution and numbers of Phase-3 Ag1000G individuals included in the analysis (right) with focus on the Far-West region (left). Classification based on Ancestry Informative Marker (AIM): FW individuals classified as “intermediate” as they show *An. coluzzii* AIM-fraction >5% and <95%. Details on population codes, AIM proportions, and total number of individuals/country are Table S1. Updated and modified from *Anopheles gambiae* 1000 Genomes, 2020. **B** - Principal component analysis based on SNPs on chromosome-X, −3 and −2. Specimens coloured by AIM-fraction. Circles = *Anopheles coluzzii* and *Anopheles gambiae*; crosses=Far-West individuals. **C** - ADMIXTURE Bayesian ancestry most parsimonious models for chromosome-X (K=3) and chromosome-3 (K=3). *Anopheles coluzzii*: BFcol (Burkina Faso), CIcol (Côte d’Ivoire), GHcol (Ghana), GNcol (Guinea), MAcol (Mali); *An. gambiae*: BFgam (Burkina Faso), GHgam (Ghana), GNgam (Guinea), GWgam (Guinea-Bissau), MAgam (Mali).

The sample data set utilised in the present study includes the genome sequences and associated metadata of 1,190 mosquito females collected at 35 sites from 7 different countries in West Africa provided by MalariaGEN Anopheles gambiae 1000 Genomes Project Phase-3 data resource^25^ (Figure 1A; Table 1). Details of the samples and associated references are provided in the Ag1000G partner studies page (https://malariagen.github.io/vector-data/studies-ag1000g.html). Only individuals from sampling locations with sample sizes >5 are included in the analysed dataset. This includes: 374 *An. coluzzii* individuals from Burkina Faso, Ghana, Côte d’Ivoire, Guinea, The Gambia, Mali; 449 *An. gambiae* individuals from Burkina Faso, Ghana, Guinea, Guinea-Bissau and Mali collected between 2004 and 2014; 274 individuals from The Gambia and 93 from Guinea-Bissau (hereafter referred to as Far-West, FW) with an uncertain species status according to the Ag1000G Consortium^4,18^, collected in 2010–2012. For the purpose of the present study, FW-populations are classified as ‘intermediate’, when the average *An. coluzzii* Ancestry-Informative Marker (AIM)-fraction of the populations is >5% and <95%. Only one population from Far West (Leibala from inland Guinea Bissau) was classified as *An. gambiae*, having an average *An. coluzzii* AIM-fraction of 2%. All other populations from Far-West (hereafter FW_pops_) were classified as intermediate.

Sequence data utilised to explore the genetic variation and structure are available from the European Nucleotide Archive (ENA; https://www.ebi.ac.uk/ena/browser/home [ebi.ac.uk] [ebi.ac.uk [ebi.ac.uk]). ENA accession numbers for the specific samples and sequencing runs used in this study are provided as Table S1. Further information on availability of these data is available from the MalariaGEN website at https://malariagen.github.io/vector-data/ag3/ag3.0.html [malariagen.github.io].”

SNPs on the three chromosomes were first filtered by the “gamb_colu” site filters to reduce sequencing and alignment error as defined by Ag1000G Consortium. A variant set/chromosome was then generated by selecting biallelic variants with a minor allele frequency ≥1% and randomly down-sampling to 100,000 SNPs per chromosomal arm. Subsequently we pruned for linkage disequilibrium by excluding variants SNPs above an r^2^ threshold of 0.01 in moving windows of 500 SNPs with a step size of 250 SNPs using the locate_unlinked function in scikit-allel Python package^26^.

To carry out genomic window-level analyses, a second biallelic SNP set was generated for the euchromatic region chromosome-3 (i.e. 34,707,855 SNPs in 3R: 1–24 Mbp; 3L: 15–41 Mbp). This region is recognized to provide the most coherent view of population structure due to the absence of polymorphic inversions^27^ and to cluster populations by geographical region rather than by *An. coluzzii* and *An. gambiae* species^4^.

Finally, SNPs included in the analysis of genomic diversity and divergence across the whole genome were chosen by selecting 191,422,813 SNPs on all chromosomes (2.3).

Table 2 reports the analyses carried out on the above datasets, which are detailed in the following paragraphs.

### 2- Genetic Variation among West African populations

Patterns of genetic variation were explored by PCA^28^, implemented by scikit-allel with the function *allel.pca (gn, n_components=10, copy=True, scaler=‘patterson’, ploidy=2)*.

Estimates of individual ancestries and detection of population structure and admixture were assessed by the maximum likelihood approach implemented in ADMIXTURE 1.3.0^29^. A 5-fold cross-validation for K values from 1 to 8 was run.

All following analyses were run on PCA inferred clusters.

### 3- Nucleotide diversity and divergence in FW

Genome-wide variation was quantified by a sliding window approach with 100,000 bp non-overlapping windows of accessible bases. After computing Fst values for all SNPs, we ranked them in descending order to identify the top 5 windows showing the highest differentiation. To associate the identified signals with their corresponding genes, we utilized the AGAP4 genome annotation following the ag3.geneset() method from the malariagen_data Python package. By mapping the genomic coordinates of the signals to known gene locations, we identified the genes located within or near the genomic regions associated with the top 5 signals.

The average pairwise nucleotide diversity (i.e., π^30^), Tajima’s D^31^, Hudson’s FST following the Bhatia et al., procedure^32^, and the absolute genetic divergence (i.e., Dxy) on 100,000 accessible bases were computed using scikit-allel^26^.

### 4- Inference of gene flow and introgression in FW clusters

The F3 statistic^33^ estimates whether allele frequency differences between the target population X and populations A and B indicates incomplete lineage sorting expected from a species tree or results from admixture. First, we tested the hypothesis that FW clusters are the result of admixture between west-African CO and GA. For a simple bifurcating tree, the product of the frequency differences (F3 (FW _pops_; CO_pops_, GA_pops_) between CO_pops_ and FW_pops_, and GA_pops_ and FW _pops_ is expected to be positive. The product can be negative only if each FWpop has equal ancestry related to both CO and GA. Thus, a significantly negative F3 value (Z<5) would provide evidence of admixture in FW population history. Second, we tested the hypothesis that a FWpop is the result of admixture between CO or GA and another FW_pop_.

After the admixture test, we conducted outgroup-F3-statistics in the form of F3 (FW_pops_, reference population; outgroup) to test shared genetic drift between FW populations and a reference population since their divergence from the outgroup.

Direction of gene-flow in the FW clusters was calculated by D-statistics^34^. *Anopheles christyi* (a non-malaria vector closely related to the *An. gambiae* complex^35^) was used as an outgroup genome following Fontaine et al^36^. Two tree topologies were ascertained with either *An. coluzzii* or *An. gambiae* as the source of gene-flow and each of the FW_pops_ together with the other of these two species as test populations as follows:

1- gene-flow between each FW_pops_ and *An. coluzzii*


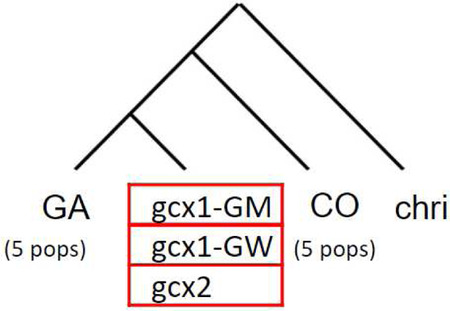


2- gene-flow between each FW_pops_ and *An. gambiae*


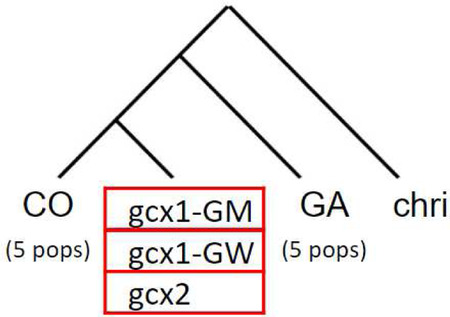


Patterson’s D statistics with a jack-knife resampling approach was applied with a block-size of 100k SNPs for each test. All the tests were done using Python package scikit-allel^26^ with the specific function “allel.blockwise_patterson_d(aca, acb, acc, acd, blen=blen)”.

TreeMix^37^ was applied to infer patterns of population splitting and build maximum likelihood trees based on correlation among allele frequencies and genetic drift. Ten TreeMix replicates were run for each m value (number of migration edges) up to 3 including -noss parameter and random seeds. To avoid converging on the same composite likelihood for each replicate, the number of SNPs per window (−k) was varied across runs from 500–1000 in 100 SNP increments using *An. christyi* as root. To evaluate the best m parameter, TreeMix output files were analysed with OptM v0.1.6^38^, which modifies the approach of Evanno et al.^39^ to choose an optimal model, using default parameters and plotted for m = 1, 2, 3 simulated models. All analyses were performed in R v3.6.3. The best tree model was plotted using the python module Toytree v.1.0^40^.

### 5- Demographic inference of West African clusters

Demographic history can provide important insights into the underlying evolutionary processes that shape genetic differentiation, such as population bottlenecks, migration and admixture. For each PCA-cluster, a site frequency spectrum (SFS) was computed using biallelic counts in SNPs from chromosome 3, using scikit-allel^26^.

Finally, to explore the demography of the gcx1-GM cluster, the diffusion approximation method of ∂a∂i^41^ was applied to analyse joint site frequency spectra. To guide development of three-population models, three different demographic models were fitted by using dadi_pipeline v3.1.5^42^ to test whether *gcx1-GM*: originated from admixture between CO and GA (admix_origin_no_mig); 2) split simultaneously to CO and GA (sim_split_no_mig); 3) experienced divergence from either CO or GA (split_nomig). Hypotheses were tested to generate a plausible demographic model, without considering gene-flow that have been assessed as described below. For all models, consecutive rounds of optimizations were performed following Portik et al.^42^. Across all analyses, the optimized parameter sets of each replicate were used to simulate the 3D-JSFS, and the multinomial approach was used to estimate the log-likelihood of the 3D-JSFS given the model. Models were compared using the Akaike information criterion (AIC), and the replicate with the highest likelihood for each model was used to calculate AIC scores and ΔAIC scores^43^. To assess the robustness of our results, the best supported model was run establishing parameter bounds to ensure that the search remains within a feasible range of values. These bounds are crucial because optimization methods often explore a wide range of parameter values, occasionally exceeding the permissible limits.

### 6- Identification of *gcx1-GM* related SNPs

To identify a set of markers that can be used downstream for taxonomic assignment, differences in Allele Frequency (DAF) between *gcx1-GM* and *An. gambiae* were computed considering only biallelic SNPs. SNPs with overall genotyping success below 10% were excluded. The percentage of *gcx1-GM*-related alleles per individual (individual allelic percentage) was computed in order to define a cut-off able to discriminate *gcx1-GM* and *An. gambiae* specimens in the far-west African region.

### 7- Haplotype phasing

Haplotypes were phased from genotypes at biallelic SNPs with a combination of read-backed and statistical phasing as recommended by the AG1000G phase 3 project. Read-backed phasing was performed on individual samples using WhatsHap version 1.0^44^.

### 8- Target-site insecticide resistance

Amino acid substitution frequencies were calculated for each population cohort using functions built into the malariagen_data python package. Predicted amino acid changes were identified based on a specified gene transcript and observed non-synonymous SNP frequencies. Frequencies were calculated for genes with an established association with target site resistance including the Voltage-gated sodium channel (*Vgsc*), the GABA-gated chloride channel subunit (*Rdl*) and acetylcholinesterase (*Ace1*), all found on chromosome two. To account for variation due to sequencing error, only substitutions present in at least one population cohort with a frequency >5% were retained.

### 9- Metabolic insecticide resistance

Genes known to be associated with metabolic resistance in *Anopheles* were targeted for Copy Number Variant (CNV) discovery. These included the CYP6AA/P cluster on chromosome two, CYP6M2 and glutathione S-transferase (GST) on chromosome three and CYP9K1 on the X chromosome. Copy number state was calculated across the genome for individuals as detailed in Lucas et al.^45^. In summary, a Gaussian HMM model was implemented to calculate coverage over 300 bp windows, normalized to account for bias in GC content. Regions with high GC content or low mapping quality were filtered. CNVs were characterized when five or more adjacent windows had a copy number state greater than two (or greater than one for males on the X chromosome). Samples with high coverage variance or CNVs with a low HMM likelihood of CNV state were removed from analyses. The proportion of individuals within each population cohort with any CNV amplification or deletion was then calculated. This frequency is based on the presence/absence of a CNV regardless of the number of copies present.

### 10- H12 Haplotype diversity statistics

We calculated the H12 measure of haplotype homozygosity across windows spanning each chromosome. The H12 statistic is described by Garud et al.^46^ and is modified from the common haplotype diversity statistic H1 to combine the first and second most common haplotype frequencies. The statistic has increased sensitivity to detect both hard and soft sweeps. To account for variation in demographic history, the window size was calibrated for each population cohort. We identified the optimal window size by plotting the distribution of H12 values across a range of window sizes and identifying the value where the 95% percentile of the H12 values was at or below 0.1. Values were plotted to identify statistical peaks suggestive of a selective sweep.

## RESULTS

### 1- Genomic structure reveals clusters in FW-Africa distinct from western *An. coluzzii* and *An. gambiae*.

Results of PCAs based on SNPs either on single chromosomes (Figure 1B, Figure S1) or from the whole genome (Figure S2A) consistently separate *gcx1*-specimens from the two clusters grouping *An. gambiae* ((GA,, including individuals from Leibala in Guinea Bissau) and *An. coluzzii* (CO) specimens, respectively. All *gcx2* individuals cluster together either between gcx1 and CO clusters (based on chromosomes -X and −3 SNPs) or close to CO (based on chromosome-2 and whole genome SNPs (Figure S2A)); i.e. CO+*gcx2*. When focusing the analysis on the centromeric region of chromosome-X - known as the CO and GA speciation island^47^– *gcx1* individuals consistently cluster separately from CO+*gcx2* and GA (Figure S2B). When focusing the analysis on the genomic regions included within chromosomal inversion 2Rd and 2La - whose frequencies characterize the so called BISSAU chromosomal form^12^ - PC1 separates individuals based on their 2La karyotype, while PC2, which explains similarly high variance, separates *gcx1* from CO+*gcx2* and GA (Figure S2C).

Results of individual-based ancestry analysis (ADMIXTURE; K=3) are largely consistent with those from PCA, suggesting a third ancestral cluster (purple) in addition to CO (red) and GA (blue) clusters (Figure 1C; Figures S3 and S4). All *gcx1* individuals show mostly “purple” ancestry, with a limited proportion of GA-ancestry on chromosome-3 in g*cx1*-GM, and some proportions of GA and CO on both chromosomes in g*cx1*-GW. On the other hand, *gcx2* same individuals show share *gcx1* and CO ancestries. At K=2, FW_pops_ and CO share the ancestry (Figures S3 and S4).

### 2- Genomic divergence of FW-clusters from *An. coluzzii* and/or *An. gambiae* in the range of interspecific divergence.

In this and in the following paragraphs results are shown from analyses on chromosome-3 euchromatic region data to assess genomic divergence and gene-flow among FW-clusters, CO and GA (including the Leibala population from inland Guinea Bissau).

To assess whether *gcx* populations form a genomic group differentiated from CO and GA, we measured the extent of divergence among clusters relative to the net genetic diversity using Hudson’s *F*_ST_ (Figure S5A). All FW-clusters show levels of differentiation with GA (*gcx1-*GM=0.032; *gcx1-GW*=0.027; *gcx2-GM*=0.035) comparable to that between species GA vs CO (0.033). Moreover, *gcx1-GM* and *gcx1-GW* show high (0.028) and intermediate (0.018) levels of differentiation with CO.

Genetic diversity statistics show: i) no significant difference in nucleotide diversity among the 3 FW_pops_, CO and GA; ii) higher values of Tajima’s D for *gcx1*-GM, suggesting lower effective population sizes compared to all other populations; iii) non-significant difference in absolute genetic divergence (*Dxy*) between FW_pops_ and CO and FW_pops_ and GA (Figure S5B)

**Figure 2.**
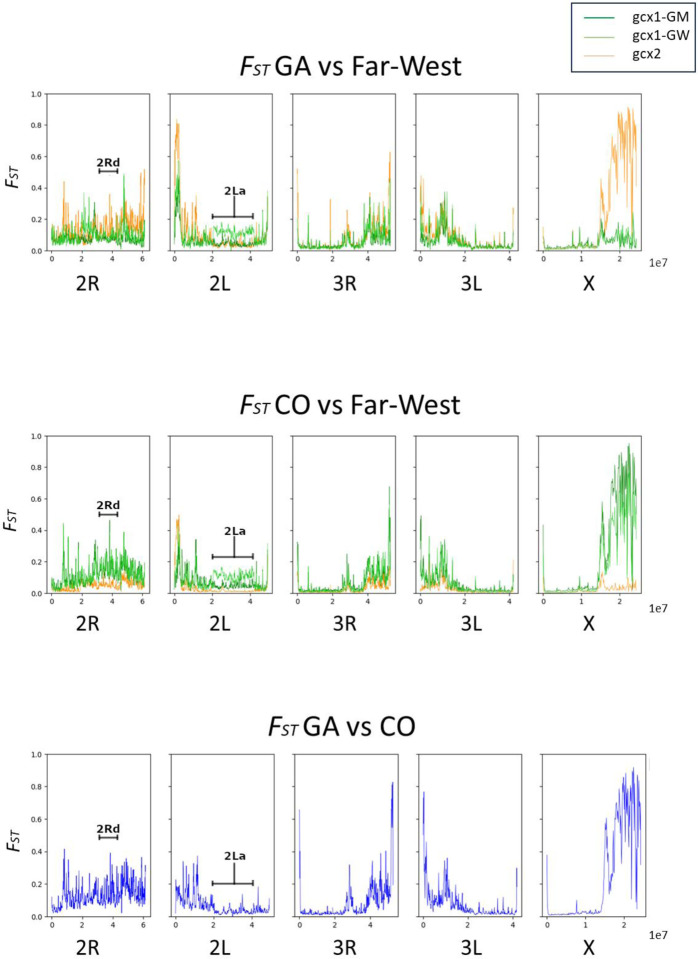
Genome-wide *F*_ST_ per 100k accessible bases between FW-clusters and *An. gambiae* (upper panel) or *An. coluzzii* (intermediate panel), and between *An. coluzzii* and *An. gambiae* (lower panel) gcx2=orange=; *gcx1-*GM=green; *gcx1*-GW=light green.

Highest differentiation is mostly observed in centromeric regions, as also occurs between the two species (Figure 2). In the centromeric region of chromosome X, high genetic differentiation is observed between *gcx2* and GA, between *gcx1*-GM and CO and between *gcx1-GW* and CO. A similar pattern is observed also in the centromeric region of chromosomal arm 3R, albeit with lower differentiation. In the centromeric region of chromosomal arm 2L (surrounding the *Vgsc* gene), high levels of differentiation are observed between the three FW-clusters and both species. Beyond the centromeric regions, the highest levels of differentiation are found in: i) the 15.1 Mbp to 15.7 Mbp region on chromosome X, between: *gcx1*-GM and CO and gcx1-GW and CO; *gcx2* and GA; GA and CO; ii) the 2La inversion region, between gcx1-GW and GA. Within the Far-West, *gcx1*-GM and *gcx1*-GW show high levels of differentiation with *gcx2* on chromosome-X and −2R centromeric regions, while virtually no differentiation is observed between *gcx1*-GM and *gcx1*-GW across the whole genome (Figure S6) with the exception of the region carrying the 2La inversion. The genes identified from the analysis of the top 5 window Fst signals are provided in Table S2. The table includes the gene symbols, genomic coordinates, and additional relevant information, such as gene function or known associations.

### 4- Patterns of genomic divergence and admixture in FW-Africa.

Genomic relationships between FW-clusters and CO and GA populations were assessed by building a population tree with TreeMix, based on genome-wide allele frequency data derived from allele counts. For this purpose, CO and GA individuals collected in different sites from the same country are grouped together as in Ag1000G and *An. christyi* is used as outgroup.

**Figure 3.**
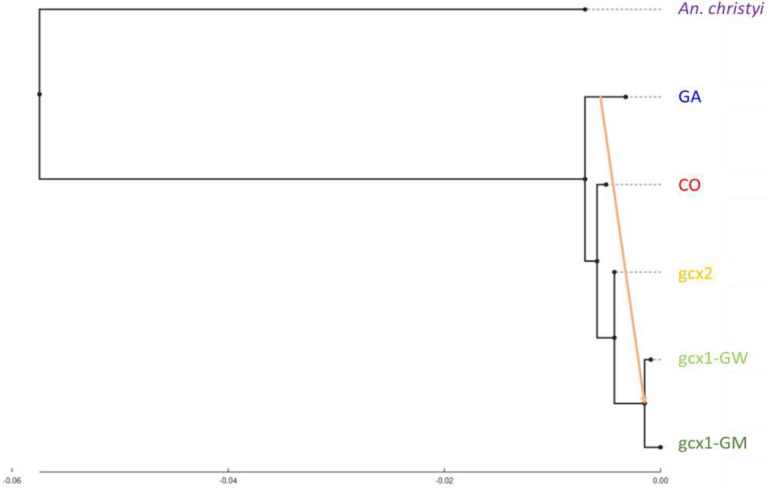
Patterns of west-African *An. gambiae* s.l. population splits and mixtures as inferred by TreeMix based on SNPs in the euchromatic region of chromosome-3. Branch lengths are proportional to the evolutionary change (the drift parameter) and terminal nodes were labelled with clusters codes. Migration edges were coloured according to migration weight.

For every m value modelled, TreeMix placed FW-clusters as sister to CO relative to GA and *An. christyi*, with a FW branch splitting from CO not long after the split from the GA lineage (Figure S7). In the optimal model (m=1 migration edge, 99% of variance explained; Figure 3; Figure S8), all FW-clusters form a clade sister to CO, and with migration from GA to *gcx1* but not to gcx2. This topology suggests there was a single FW ancestral population similar to CO, with subsequent divergence between *gcx1* and *gcx2* driven in part by GA introgression into *gcx1*, leaving *gcx2* retaining more affinity to CO. Models with larger m values have higher likelihoods, but not sufficiently so to justify the more complex topology, and are largely congruent with the optimal model. For example, with m=2, *gcx2* has ancestry from both a CO-like branch and a *gcx1*-like branch, but it remains true that all FW clusters share a common ancestry and gcx2 is closer to CO than *gcx1* is. This interpretation is largely consistent with scenarios supported by alternative methods mentioned above like principal component analysis and ADMIXTURE.

To evaluate gene-flow and admixture between FW_pops_ and CO- or GA-clusters, F3- and Patterson’s D statistics were performed.

Results of admixture-F3 tests performed to evaluate the hypothesis that FW_pops_ are the result of admixture between west-African CO and GA, reject this hypothesis (Z-values >> 5 in all 75 tests; Table S3). On the other hand, results of the second F3 analysis testing the hypothesis of admixture between West African CO and FW_pops_ suggest that *gcx1*-GW might have originated from admixture between *gcx1*-GM and either *gcx2* or CO (Figure S9). Outgroup-F3 was performed to calculate shared genetic drift of FW_pops_ and either CO or GA as reference populations and showed a closer relationship of FW_pops_ with CO (F3 = 0.018–0.030).

We performed Patterson’s D statistics to test for excesses of shared derived polymorphisms. Results from all combinations of tests among FW_pops_ and either CO or GA showed statistically significant levels of gene-flow between each of the three FW_pops_ and CO (D<0; Z score>−5), but a lack of gene flow with GA (D>0; Z score<5). Rare occurrence of gene-flow is also observed between CO and GA populations (Table S4).

### 5- Demographic history in West Africa

**Figure 4.**
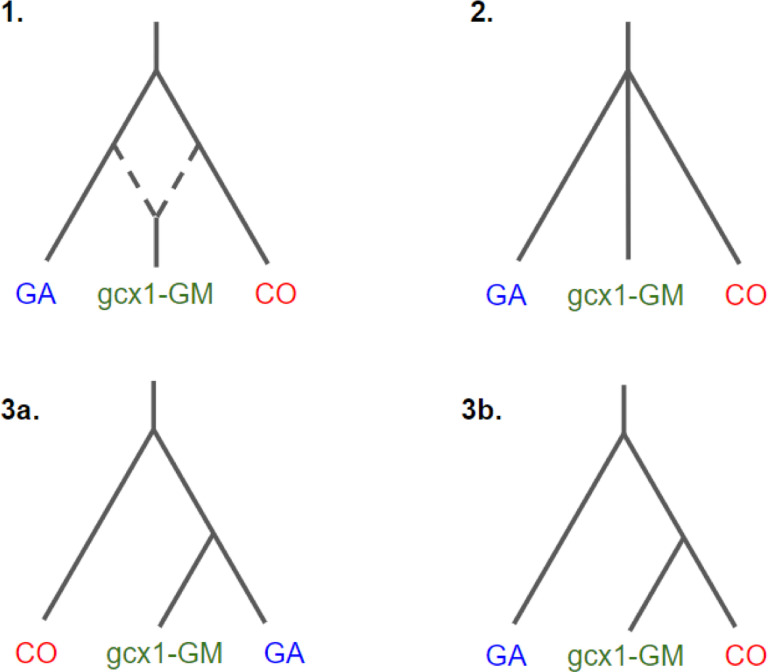
Demographic models explored by ∂a∂i analysis: 1) Admixed origin model; 2) Simultaneous split model, the best supported model depicted by the analysis; 3) Simple split model with *gcx1*-GM stemming from GA (3a) or CO (3b).

Folded allele frequency spectra results show that FW-clusters have an excess of rare variants, suggesting a population expansion equivalent to that observed in CO and GA (Figure S10). We here focused on inferring the demography of *gcx1*-GM cluster, previously shown results for which suggest may be isolated from CO and GA. To this aim, ∂a∂i analysis was performed with the goal to discriminate among three alternative hypotheses , i.e. whether *gcx1*-GM 1) originated from admixture between CO and GA; 2) split simultaneously to CO and GA; 3) experienced divergence from either CO or GA (Figure 4). Lowest AIC values support the second hypothesis (Table S5, θ = 881.21; ΔAIC ≥ 82,840 for all other models) suggesting that the divergence of the *gcx1*-GM population is contemporary to the split between CO and GA.

### 6- Identification of *gcx1*-GM related SNPs

*Anopheles coluzzii/An. gambiae* AIMs separate *gcx1*-GM from CO^4,48^ and *gcx2* (see Figure 1). In order to design an approach to be exploited for *gcx1*-GM and GA discrimination, we focused the analysis on allele frequency differences (DAF) between them (Table S6). Three fixed SNPs are identified on chromosome-2 and the threshold >98% used for *An. coluzzii/An. gambiae* -specific AIMs allows the identification of a further three SNPs (all on chromosomal arm 2R). Arbitrarily, lowering the threshold to >85% identified 201 *gcx1*-GM-related SNPs, 85% of which are on chromosome-2 and 1.5% on chromosome-X. Almost all of the identified chromosome-2 SNPs are clustered in two regions on chromosome arm 2R (15% within 28.01–28.53 Mb; 45% within 47.79–47.85 in Mb) and in two regions on chromosome arm 2L (16% within 1.95– 2.53Mb; 17% within 46.60– 46.74 Mb). Figure S11 shows the location of chromosome-2 *gcx1*-GM-related SNPs on a graph showing chromosome-2 FST per 1bp between *gcx1*-GM and *An. gambiae*. Table S6 also reports the genes included in these four chromosomal regions.

**Figure 5.**
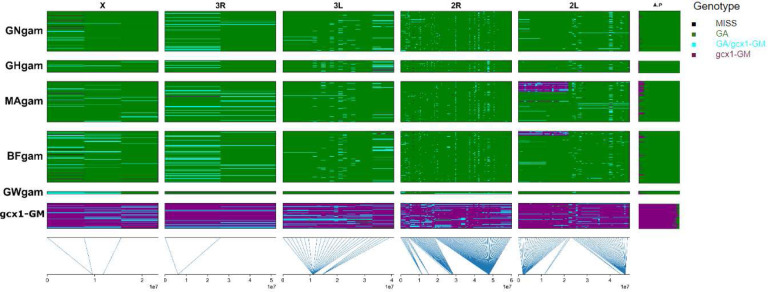
*gcx1*-GM vs *Anopheles gambiae* markers plot. Rows represent individual mosquitoes (grouped by population) and columns represent SNPs (grouped by chromosome arm). Approximate chromosomal position is given below the graph. Colours represent *gcx1*-GM related (Purple), *An. gambiae* (GA; Green), *gcx1*-GM/GA (cyan) heterozygous genotypes; Missing genotypes in black. The column at the far right shows the individual allelic percentage (A.P.).

Computation of individual allelic percentages (Figure 5) shows that 74 out of 77 *gcx1*-GM specimens carry >80% of the 201 *gcx1*-GM-related alleles, while all 449 GA specimens carry <20% of *gcx1*-GM-related alleles, suggesting these values as possible cut-offs for the individual allelic percentage to discriminate *gcx1*-GM from GA with >99.4% accuracy.

Ranges of individual allelic percentages of the above *gcx1*-GM related alleles for the other clusters are: i) 6–60% for all CO; ii) 31–90% for all *gcx2* and iii) 61–80% for 16 out of 93 *gcx1-*GW specimens, with the remaining 77 specimens showing values >80%. Thus, no CO would be falsely identified as *gcx1*-GM exceeding an 80% cut-off, and no *gcx2* or *gcx1*-GW would be falsely identified as GA under a 20% cut-off, though some would be identified as *gcx1*-GM (Figure S12).

### 7- Insecticide resistance in FW-_pops_

Amino acids changes at target sites known to be associated to insecticide resistance (i.e. *Vgsc*, *Rdl* and *Ace-1*) are virtually absent in FW_pops_, although they are commonly observed – sometimes at very high frequencies - in all Phase-3 CO and GA populations analyzed (Figure S13).

Analogously, CNVs associated with metabolic insecticide resistance are common in CO and GA populations, but are either absent (detoxification gene GST) or present at much lower frequencies (CYP6AA1, CYP6AA2, CYP6AA/P, CYP6M2 and CYP9K1) in FW_pops_ (Figure S14). Both *gcx1-*GM and *gcx1-*GW lack evidence of CNVs associated with insecticide resistance. Although an amplification frequency of up to 57% was observed at CYP9K1, the presence of a duplication was not accompanied by a supporting signal of selection based on the H12 scans (see below), unlike for other species. CYP6AA1, CYP6AA2 (overexpressed in pyrethroid resistant populations) and CYP9K1 was observed at low frequencies (1–7%) in *gcx2*.

In contrast to what was observed in CO and GA, results from the H12 haplotype diversity selection scans show negligible evidence of positive selection in the vicinity of IR amino acid substitutions across the genome in FW-_pops_. In *gcx1*-GM the only selective peak is observed at CYP6AA/P and is not associated with any amino acid change at the locus (see above). In gcx2, two selective peaks are observed at *Rdl* and around the CYP9K1 gene (Figure S15).

## DISCUSSION

Analysis of Phase-3 Ag1000G populations from West Africa suggests the existence of a demographically stable novel cryptic taxon of the *An. gambiae* complex in the coastal region of far-west Africa. This taxon is best represented by the *gcx1*-GM population, and it is hereafter provisionally named Bissau molecular form, due to the possible partial overlap with the BISSAU chromosomal form (see below). Several lines of evidence support the hypothesis that this taxon is distinct from any described species *sensu stricto*. In The Gambia, the Bissau molecular form is characterized by: i) a unique ancestral gene-pool (purple in ADMIXTURE analysis; Figure 1) and genetic isolation from West-African CO and GA (as revealed by PCA results); ii) levels of divergence from West-African CO (*F*_ST_=0.032) and GA (*F*_ST_=0.028) in the range of that observed between the two species (*F*_ST_=0.033); iii) GA-like AIMs but with differentiation from GA by 201 SNPs mainly on chromosome-2 close to fixation (i.e. showing differences in allele frequencies >85%). Notably, results from admixture-F3 statistics suggest that the Bissau molecular form did not originate from admixture between CO and GA, but rather diverged at the same time of their divergence (as revealed by ∂a∂i).

In coastal Guinea Bissau, the Bissau molecular form seems to have undergone admixture, leading to a demographically stable population (corresponding to *gcx1*-GW), which: i) clusters with *gcx1*-GM in PCA and is characterised by the prevalence of GA-AIMs; ii) is characterized by admixed Bissau molecular form and *gcx2*-ancestries (as revealed by ADMIXURE with K>3); iii) shows a high level of divergence with GA (*F*_ST_=0.027) and intermediate ones with CO (*F*_ST_=0.018). F3 analysis suggests an origin of this population from an admixture between Bissau molecular form and coastal CO-populations (including *gcx2*). Closeness to *gcx1*-GM and gene-flow with CO is also confirmed by Patterson’s statistics (Table S4).

In The Gambia, the Bissau molecular form is found in sympatry with *gcx2* (hereafter FW-*An. coluzzii*), which is a distinct CO-like population (as revealed by PCA, ADMIXTURE, CO-like AIMs, F3) showing high levels of differentiation from GA (Fst=0.035). Interestingly, further evidence of the presence of FW-*An. coluzzii* at the eastern extreme of the country (not sampled in the present study) come from Ag1000G Phase3.5 dataset (Figure S16).

Results from Tree-mix suggest that all the FW populations originated from a single *An. coluzzii* -like FW ancestral population. This was followed by the divergence between Bissau molecular form and FW-*An. coluzzii*, driven in part by ancestral introgression of *An. gambiae* into the Bissau form, leaving FW-*An. coluzzii* retaining more affinity to *An. coluzzii*. Patterson’s statistics results suggest recent gene-flow between all FW populations and *An. coluzzii*.

It is relevant to note that while most analyses were carried out on the euchromatic region of chromosome-3 - which is well known to cluster individuals by geographical region, rather than to be associated with interspecific divergence between *An. coluzzii* and *An. gambiae* ^4^- genomic structure analyses (PCA and ADMIXTURE) were carried out on the whole genome, on each the three chromosomes and on specific chromosomal regions separately, providing consistent results. The first specific region is the pericentromeric region of chromosome-X (Figure S2B), a region of reduced recombination known to be associated with reproductive isolation between *An. coluzzii* and *An. gambiae*^49^. The second includes the euchromatic regions within 2Rd and 2La paracentric inversions of chromosome-2 (Figure S2C), which have been included in the analysis, as differences in the frequencies of the two inversion polymorphisms were associated with BISSAU chromosomal form in the same geographic regions^12,24^. PCA results on SNPs within the two inversions suggest a major role of these genomic regions in separating the Bissau molecular form from *An. coluzzii*, FW-*An. coluzzii* and *An. gambiae*. This latter result suggests a possible partial overlap between the BISSAU chromosomal form and the Bissau molecular form, analogous to the relationship between the SAVANNA and MOPTI chromosomal forms and the S- and M-molecular forms, known today as *An. gambiae* and *An. coluzzii*.

The actual existence of the Bissau molecular form as a novel cryptic taxon within the *An. gambiae* complex is further supported by lack of genomic evidence of insecticide resistance mechanisms commonly observed in *An. gambiae* and *An. coluzzii* across their entire range, sometimes also at very high frequencies. First, amino acid changes associated with target site resistance mechanisms (i.e. *Vgsc*, *Rdl* and *Ace1*) are virtually absent in all FW-pops (Figure S13). Analogously, CNVs associated with metabolic insecticide resistance are common in *An. coluzzii* and *An. gambiae* populations, but are either absent (detoxification gene GST) or present at much lower frequencies (CYP6-related) in FW-pops (Figure S14). Finally, H12 haplotype diversity selection scans show negligible evidence of positive selection in the vicinity of IR amino acid substitutions. This was already reported by Clarkson et al.^50^ for fewer FW-populations (Ag1000G Phase-2), despite lack of evidence of lower insecticide pressure in coastal FW-region compared to inland western regions. These genomic results, not only support the ancestral divergence of the Bissau molecular form from the two species, but also suggest that the novel taxon may have different insecticide resistance mechanisms. In fact, the analysed specimens were collected indoors, suggesting that the Bissau molecular form is likely to be exposed to malaria vector control interventions by either LLIN or IRS, commonly implemented in the FW-region as well as across sub-Saharan Africa.

Interestingly, recent studies on populations collected in The Gambia few (>4 years) years after those analysed in the present work report presence of the 995F *kdr* resistant allele in *An. coluzzii*, in *An. gambiae* and in putative hybrids, both in the coastal area (frequencies up to 17%) and inland eastern sites (frequencies up to 28%)^51^. This may suggest adaptive introgression in the Bissau molecular form (as extensively shown between *An. coluzzii* and *An. gambiae*^10,52,53^), but lack of genomic identification of the sampled individuals precludes confirming this speculation. In fact, conventional species identification by IGS markers provides indications of “unusual” situations when IGS heterozygous patterns are observed (as in the Far-West region), but does not allow discrimination between *An. gambiae* and Bissau molecular forms individuals. The SNPs identified as distinctive of the Bissau form will be instrumental for the development of a multiplex assay for the form identification, which would allow clarification of this point, as well as to study the form’s ecology, behaviour and epidemiological significance.

It is not the first time that a novel putative cryptic taxon has been found in the *An. gambiae* complex following observations of unusually high frequencies of individuals characterised by polymorphic *An. coluzzii/ An. gambiae* IGS diagnostic markers. In Burkina Faso, a subgroup genetically distinct from sympatric *An. coluzzii* was observed and named GOUNDRY^54–56^. Subsequently a second group genetically divergent from *An. coluzzii* and all other *An. gambiae* complex species (named *Anopheles TENGRELA*) was observed in the same geographic region and GOUNDRY was shown to be a recently diverged population from the TENGRELA lineage via introgression from *An. coluzzii*^57^. However, the present report is unique from several perspectives. First, it is the first time that adult field females are suggested to belong to a new taxon close to *An. coluzzii* and *An. gambiae*, as both TENGRELA and GOUNDRY were collected exclusively as larvae and no adults have ever been found despite extensive and long-term efforts. Second, present results suggest that the size of the geographic range of the Bissau molecular form may extend far beyond that of TENGRELA and GOUNDRY. Third, several recurrent observations support the presence of stable populations with unique gene-pools in the field (likely corresponding to the Bissau molecular form): i) admixed *An. gambiae* populations observed in coastal The Gambia since 2005^20,58^; ii) frequencies >20% of individuals characterized by *An. coluzzii/An. gambiae* IGS genotypes observed in coastal Guinea Bissau since 1995^10,19,58^; iii) data on chromosome −X and −3 microsatellites and on chromosome-2R paracentric inversion polymorphisms showing that these admixed coastal *An. gambiae* populations were highly differentiated from *An. gambiae* populations from inland areas both in The Gambia^21^ and in Guinea Bissau^23^.

## CONCLUSIONS

The results presented lead to a change of perspective on the major malaria vector species at the extreme west of their range, supplanting previous hypotheses of either species radiation promoted by massive genomic introgression between *An. coluzzii* to *An. gambiae* (based on the genotyping of few species-specific markers^22,23^), or of an “intermediate” far-west taxon (based on WGS data from Phase-1 Ag1000G dataset; Miles et al. 2017). At this stage it is not possible to assess the possible implications of this change of perspective on malaria transmission and conventional vector control in the region. However, it is already evident that the existence of a novel possibly widespread putative taxon in addition to those representing the actual target of future gene-drive base control interventions^59,60^, will represent a new challenge for these innovative mosquito control approaches.

There is still much to understand before defining the taxonomic status of the Bissau molecular form and its hybridization and introgression with sympatric *An. coluzzii* and *An. gambiae* populations. The possibility to couple PCR-genotyping of *An. gambiae* and *An. coluzzii* AIMs^46^ with a multilocus genotyping approach to be developed based on the form-specific SNPs identified in the present work (and to be validated on a wider collection of samples) opens the possibility to identify Bissau form individuals at all stages of its life-cycle. This will allow characterization of its geographical range, its bionomics and, most importantly, its epidemiologically-relevant traits.

## Supplementary Material

1

## Figures and Tables

**Table 1.** Phase-3 west-African Ag1000G population codes, AIM proportions, and total number of individuals/country. Species are identified based on Ancestry Informative Marker (AIM) on all chromosomes except chromosomal arm 2L. Intermediate populations are characterized by an average *An. coluzzii* AIM-fraction >5% and <95% .

Taxon	Country	Population	N° Locations	AIM fraction	Total
*An. gambiae*	Burkina Faso	BFgam	4	0.02	157
	Ghana	GHgam	2	0.02	36
	Guinea	GNgam	2	0.02	123
	Guinea-Bissau	GWgam	1	0.02	8
	Mali	MAgam	5	0.02	125
*An. coluzzil*	Burkina Faso	BFcol	3	0.98	135
	Cote d’Ivoire	CIcol	1	0.97	80
	Ghana	GHcol	3	0.97	63
	Guinea	GNcol	1	0.96	11
	Mali	MAcol	7	0.98	85
Intermediate	Guinea-Bissau	gcx1-GW	2	0.20	93
	The Gambia	gcx1-GM	2	0.09	77
		gcx2	3	0.92	197

**Table 2.** *Anopheles coluzzii* and *An. gambiae genomic* regions and SNPs analysed and analytic approaches applied.

Region	N° of SNPs	Analyses
**Chromosome-X**	91,125	PCA, ADMIXTURE
**Chromosome-X centromere** (15–24 MB)	8,885	PCA
**Chromosome-2**	137,428	PCA
**Chromosome-2, 2Rd inversion** (33.57–41.36 MB)	35,467	PCA
**Chromosome-2, 2La inversion** (20.52–42.16 MB)	57,230	PCA
**Chromosome-3**	160,926	PCA, ADMIXTURE, TreeMix
	34,707,855	F3, Patterson’s D, SFS, *F*_ST_ and diversity statistics (*Dxy,* Tajima’s D, pi, Watterson’s Theta), Dadi
**Whole genome**	191,422,813	Genome-wide *F*_ST_

## Data Availability

The sequencing and variation data utilised in this study are part of the MalariaGEN Anopheles gambiae 1000 Genomes Project Phase-3 data resource. Sequence data are available from the European Nucleotide Archive (ENA; https://www.ebi.ac.uk/ena/browser/home [ebi.ac.uk] [ebi.ac.uk [ebi.ac.uk]). ENA accession numbers for the specific samples and sequencing runs used in this study are provided as TableS1. Further information on availability of these data is available from the MalariaGEN website at https://malariagen.github.io/vector-data/ag3/ag3.0.html [malariagen.github.io].”
