# Supplementary material for: Speciation within the Anopheles gambiae complex: high-throughput whole genome sequencing reveals evidence of a putative new cryptic taxon in ‘far-west’ Africa
Source: Res Sq. 2024 Mar 18:rs.3.rs-3914444. Preprint. [Version 1] doi: 10.21203/rs.3.rs-3914444/v1 (PMC10984024; doi:10.21203/rs.3.rs-3914444/v1)
Supplement: 1 [file NIHPPrs3914444V1-supplement-1.pdf]

SUPPLEMENTARY TABLE LEGENDS

Table S1. Metadata of Ag1000G Phase3 *Anopheles gambiae* s.l. samples from West Africa, including specimens ID, information about sampling country, year (Y) and month (M), location (Site, Longitude and Latitude), gender (G), AIM An. coluzzii fraction based on Ancestry Informative Markers (AIMs) on all chromosomes except chromosomal arm 2L, species characterization by AIMs, population ID based on country and taxon and Ena run accession ID. NA=not available

Table S2. Top 5 diverging genes depicted by Fst statistics for six comparisons: *gcx1*-GM vs CO, *gcx1*-GM vs GA, *gcx1*-GW vs CO, *gcx1*-GW vs GA, *gcx2* vs CO and *gcx2* vs GA. Each row corresponds to a specific gene, providing information on chromosome location, gene position, description, strand orientation, window signal position, and Window Fst values.

Table S3. Results of F3-statistics of comparisons among either PCA-clusters, FWpops, or West African populations. The table reports chromosome information, test type, F3-statistics values, standard errors (SE), and Z-scores (highlighting high statistical values with Z< -5 in bold).

Table S4. Results from Patterson's D statistics, among either PCA-clusters or between Far-West and West African populations. The table shows chromosome information, test type, D-values, standard errors (SE), and Z-scores. Dark grey highlight = results with negative D and significant Z score. Light-grey highlight = results with Positive D and significant Z score**.**

Table S5. Results from best models tested by Diffusion Approximations for Demographic Inference (∂a∂i), evaluating log-likelihood, Akaike Information Criterion (AIC), chi-squared values, estimated theta, and optimized parameters for different demographic models.

Table S6. Location and details of all SNPs selected for their allele specificity between *An. gambiae* and *gcx1*-GM.

SUPPLEMENTARY FIGURE LEGENDS

Figure S1. Variance Explained by Principal Component Analysis of Phase-3 Ag1000G *Anopheles gambiae* s.l. from West and Far-West Africa based on SNPs on chromosome-X (A), -3 (B) and -2 (C).

Figure S2. Results of Principal Component Analysis (and variance explained) of Phase-3 Ag1000G *Anopheles gambiae* s.l. from West and Far-West Africa based on SNPs either on: A - all chromosomes (100k SNPs for each chromosomal arm), B - chromosome-X centromeric island (8,885 SNPs), or C - chromosome-2 inversion 2Rd (57,330 SNPs) and 2La (35,467 SNPs) regions. Specimens coloured by AIM-fraction. Circles =CO and GA; crosses=FW-individuals.

Figure S3. ADMIXTURE analysis of Phase-3 Ag1000G *Anopheles gambiae s.l.* from West and Far-West Africa genomic structure based on 160,926 SNPs on euchromatic region of chromosome-3. A - Line graph of CV error values for each ancestry model denoted by K. B Ancestry proportions for ADMIXTURE models from K=2 to K=8 ancestral populations. Each vertical bar represents the proportion of ancestry within a single individual, with colours corresponding to ancestral populations.

Figure S4. ADMIXTURE analysis of Phase-3 Ag1000G *Anopheles gambiae* s.l. from West and Far-West Africa genomic structure based on 91,125 SNPs on chromosome-X region. A - Line graph of CV error values for each ancestry model denoted by K. B Ancestry proportions for ADMIXTURE models from K=2 to K=8 ancestral populations. Each vertical bar represents the proportion of ancestry within a single individual, with colours corresponding to ancestral populations.

Figure S5. Genetic divergence and diversity among Far West clusters (*gcx1*-GM, *gcx1*-GW, *gcx2*) and western *Anopheles gambiae* (GA) and *An. coluzzii* (CO) clusters in the euchromatic region of chromosome-3. A - Pairwise Fst scatterplot showing levels of divergence; **B-** Genetic diversity statistics: π, Tajima's D, Watterson theta, *Dxy*.

Figure S6. Genome-wide F_{ST} among Far West *Anopheles gambiae* s.l. clusters (*gcx1*-GM, *gcx1*-GW, *gcx2*)

Figure S7. Patterns of population splits and mixtures in the history of Far West *Anopheles gambiae* s.l. clusters (*gcx1*-GM, *gcx1*-GW, *gcx2*) by TreeMix analysis based on chromosome-3 euchromatic region (up to 3 migration events).

Figure S8. Summary statistics of TreeMix analysis produced by OptM up to 5 migration edges. (a) mean and standard deviation (SD) across 10 iterations for the composite likelihood L(m)(Panel 1). (b) proportion of variance explained. All models are above the 99.8% that recommended by Pickrell and Pritchard. (c) second-order rate of change (Dm) across values of m.

Figure S9. Error plots from Admixture f3 analysis performed to test the hypothesis that FWpops are the results of admixture between west-African CO and GA.
Red bar shows statistically significant tests (i.e. Z< -5). Blue bar shows a not statistically significant test (i.e. Z > -5).

Figure S10. Folded Site frequency spectra of Far West clusters (*gcx1*-GM=green; *gcx1*-GW=lime green; *gcx2*=orange) and western *Anopheles gambiae* (blue) and *An. coluzzii* (red) clusters.

Figure S11. Location of chromosome-2 *gcx1*-GM-related SNPs (in red) on Manhattan Plot of Fst values per 1bp between *gcx1*-GM and *An. gambiae*.

Fig S12. Far-west populations vs *An. coluzzii* and *An. gambiae* markers plot.

Figure S13. Amino acids change frequencies on known target-site insecticide resistance genes (i.e. Vgsc, Rdl and Ace-1) in Far West clusters (*gcx1*-GM; *gcx1*-GW; *gcx2*) and western *Anopheles gambiae* and *An. coluzzii* populations.

Figure S14. Copy Number Variation (CNV) frequencies in genes known to be associated to metabolic insecticide resistance in Far West clusters (*gcx1*-GM; *gcx1*-GW; *gcx2*) and western *Anopheles gambiae* and *An. coluzzii* populations.

Figure S15. Genome scans for signature of recent selection in Far West clusters (*gcx1*-GM; *gcx1*-GW; *gcx2*) along the 3 chromosomes.
